# Thermally Activated Delayed Fluorescence in Cu^I^ Complexes Originating from Restricted Molecular Vibrations

**DOI:** 10.1002/chem.201701862

**Published:** 2017-08-10

**Authors:** Guangfu Li, Roberto S. Nobuyasu, Baohua Zhang, Yun Geng, Bing Yao, Zhiyuan Xie, Dongxia Zhu, Guogang Shan, Weilong Che, Likai Yan, Zhongmin Su, Fernando B. Dias, Martin R. Bryce

**Affiliations:** ^1^ Institute of Functional Material Chemistry Faculty of Chemistry Northeast Normal University Renmin Street No. 5268 Changchun 130024 P. R. China; ^2^ Physics Department Durham University Durham DH1 3LE UK; ^3^ State Key Laboratory of Polymer Physics and Chemistry Changchun Institute of Applied Chemistry Chinese Academy of Sciences Changchun 130022 P. R. China; ^4^ Chemistry Department Durham University Durham DH1 3LE UK

**Keywords:** aggregation-induced emission, copper, Cu(I) complexes, photophysical properties, thermally activated delayed fluorescence

## Abstract

The mechanism of thermally activated delayed fluorescence (TADF) in molecules in aggregated or condensed solid states has been rarely studied and is not well understood. Nevertheless, many applications of TADF emitters are strongly affected by their luminescence properties in the aggregated state. In this study, two new isomeric tetradentate Cu^I^ complexes which simultaneously show aggregation induced emission (AIE) and TADF characteristics are reported for the first time. We provide direct evidence that effectively restricting the vibrations of individual molecules is a key requisite for TADF in these two Cu^I^ complexes through in‐depth photophysical measurements combined with kinetic methods, single crystal analysis and theoretical calculations. These findings should stimulate new molecular engineering endeavours in the design of AIE–TADF active materials with highly emissive aggregated states.

Recent developments of thermally activated delayed fluorescence (TADF) materials have attracted tremendous attention as they can be used as highly efficient emitters for organic light emitting diodes (OLEDs).^1^ TADF is a promising mechanism for converting electricity into light with an internal quantum efficiency (IQE) of nearly 100 %, which utilizes the up‐conversion from triplet excitons to singlet states by reverse intersystem crossing (RISC).^2^ However, a stubborn problem for TADF materials in practical applications is that most TADF emitters suffer from aggregation‐caused quenching (ACQ) in the condensed phase. Therefore, effective TADF can be realized in a doped host–guest system only with careful control of their concentrations to restrict the nonradiative deactivation processes. Aggregation‐induced emission (AIE) materials are, therefore, able to respond to these challenges. AIE molecules were first reported by Tang et al,^3^ and while they show weak emission in dilute solution, upon aggregation they emit strongly, due to restriction of intramolecular rotation. Recently, Yasuda et al. reported a series of *o*‐carborane derivatives that simultaneously show AIE and TADF characteristics.^4^ OLEDs utilizing these molecules as a nondoped emission layer exhibited maximum EQEs as high as 11 %. Thus, AIE–TADF active materials are promising luminescent materials and are widely used in numerous optoelectronic applications especially for non‐doped OLEDs. However, the mechanism of TADF in aggregated molecules is still unclear and has not been studied in detail, although it is essential for many of the desired applications of these emitters. Fundamental factors that lead to enhanced emission of dyes in viscous and solid‐state environments are of ongoing interest.^5^

Cu^I^ complexes with TADF characteristics have been used in OLEDs and some derivatives show impressive electroluminescent performance.^6^ Recently, dual‐emissive Cu^I^ complexes that can simultaneously show TADF and phosphorescent emission have been demonstrated.^7^ Cu^I^ complexes have the practical advantages of the relatively low‐cost and high abundance of copper, in contrast to rare metals such as iridium.

The aim of the present study is to explore the coexistence of TADF and AIE in the new copper complexes **1** and **2**. We demonstrate that both complexes show almost no emission in solution, but strong luminescence and obvious TADF is observed with an extremely short delayed lifetime in their aggregated state. To our knowledge, these combined photophysical phenomena have not been previously reported in Cu^I^ complexes, although the coexistence of both TADF and AIE in all‐organic molecules has been reported in a few cases.^8^ The relationship between AIE and TADF is still not clear. Herein, we address the pertinent question: does aggregation induce or promote TADF? This topic is of fundamental importance for a better understanding of the mechanism of AIE–TADF and further designing this kind of material for use in OLEDs. The kinetics of TADF are investigated in two Cu^I^ complexes in pristine film state and doped in PMMA matrices. The PMMA matrices of different molecular weight, doped with complexes **1** and **2**, have been used in solid‐state “dilution” experiments to probe the role of intramolecular vibrations on the observation of TADF. The similar TADF behavior presented in both high molecular weight host (H‐PMMA) and in pristine films clearly reveals that effectively restricting specific vibrations of individual emitter molecules is the origin of TADF for these Cu^I^ complexes. Thus, a guideline for designing AIE–TADF active materials is presented.

The chemical structures of two new isomeric complexes **1** and **2** are shown in Figures 1a and 1b. Their synthesis and characterization data are presented in the Supporting Information. The UV/vis absorption and emission spectra of **1** and **2** in degassed solution (CH_2_Cl_2_) and pristine film are depicted in Figure S6 (Supporting Information). Upon photoexcitation, **1** and **2** are almost non‐emissive in pure CH_2_Cl_2_ solution at room temperature. However, bright emission is observed in the pristine film state with a short luminescence decay time. This short‐lived luminescence is in contrast with conventional Cu^I^ complexes, where luminescence is observed with relatively long decay times (phosphorescence decay times of several 100 μs up to a few ms).^9^ The luminescence decays of complexes **1** and **2** in their pristine film state are even shorter than the recently reported decay times in mono‐nuclear Cu^I^ complexes with TADF.2c, 6a–6c, 7 This implies that **1** and **2** may be outstanding TADF materials with high reverse intersystem crossing rate (*k*_RISC_) by efficiently eliminating the triplet–triplet annihilation and triplet–polaron quenching in their pristine film state.^9^ The detailed photophysical data of **1** and **2** are summarized in Table S1. The energy levels of the complexes calculated from their electrochemical and photophysical data are listed in Table S2.

**Figure 1:**
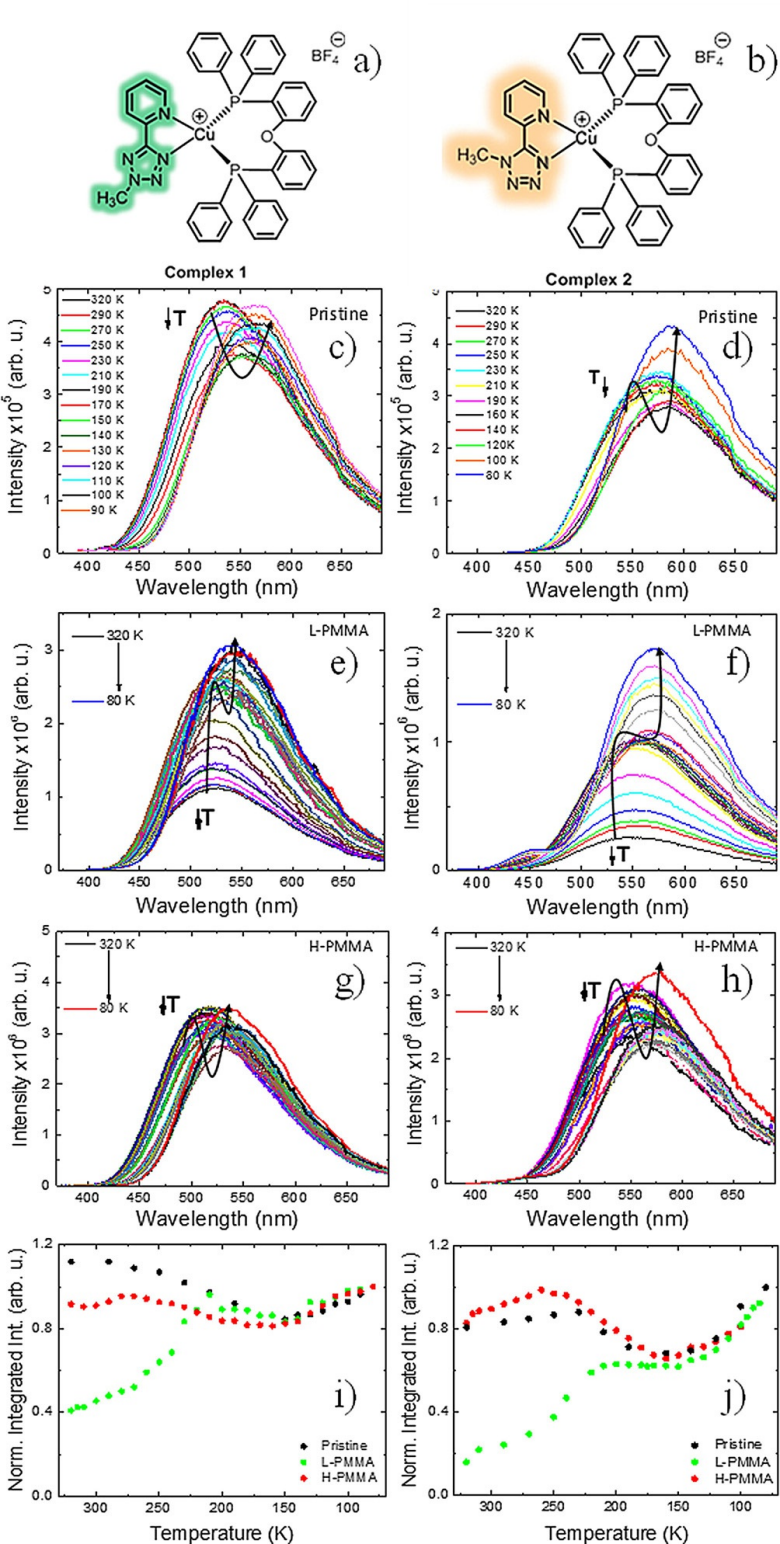
Chemical structures of isomeric complexes a) **1** and b) **2**. The steady‐state fluorescence from 320 to 80 K in pristine film for c) **1** and d) **2**, respectively. The steady‐state fluorescence from 320 to 80 K in l‐PMMA for e) **1** and f) **2**, respectively. The steady‐state fluorescence from 320 to 80 K in H‐PMMA for g) **1** and h) **2**, respectively. Temperature dependence of the integrated luminescence intensity as a function of temperature (K) for i) **1** and j) **2**, integrated intensity normalized at 80 K.

To investigate the effect of molecular vibrations on the emission of **1** and **2** in their aggregated state, steady‐state and time‐resolved photoluminescence experiments were performed in their pristine film state, and in composite films of both complexes dispersed in PMMA hosts. Two PMMA samples, one with low molecular weight, l‐PMMA (Mn=15,000), and the other with high molecular weight, H‐PMMA (Mn=960,000), were used to fabricate thin films of **1** and **2**. Thermal gravimetric analysis (TGA) data showed that H‐PMMA has a significantly higher Tg (130 °C) than l‐PMMA (86 °C) consistent with H‐PMMA being a stiffer material. The temperature dependence of the steady‐state emission spectra in these films is compared with the temperature dependence of the emission in pristine films of **1** and **2** that are used as references. This allows the roles of thermal vibrations and intermolecular interactions on the excited state dynamics in these two complexes to be determined.

The two PMMA hosts with different molecular weight appear to be able to suppress molecular vibrations to different degrees. This results in marked differences of the temperature dependence of the photoluminescence of **1** and **2**, collected in pristine films: Figures 1 c and 1d; in l‐PMMA Figures 1 e and 1f; and in H‐PMMA Figures 1 g and 1 h. Additionally, in Figures 1 i and 1j the temperature dependence of the photoluminescence in the three samples shows clear differences in the temperature region above 170 K, but agree extremely well at temperatures below 170 K.

In the pristine film, the photoluminescence spectra of **1** and **2** show a dominant TADF mechanism between 320–170 K (Figures 1 c and 1d). In this temperature range the emission intensity first increases and then decreases with decreasing temperature, showing a typical bell‐shape that is characteristic of the equilibrium between the singlet and triplet states, mediated by the *k*_RISC_, which drives the up‐conversion of triplets back to the singlet manifold, and the thermal vibrations (internal conversion: IC) that quench the excited state population.^10^

Below 170 K, the luminescence intensity is dominated by phosphorescence from the lowest T_1_ state, and is thus controlled by the decrease in the internal conversion, and as this continues decreasing with temperature, the steady‐state emission slowly increases as observed in Figure 1i). Note also the change observed in the emission spectra in Figures 1c and 1d. With decreasing temperature *λ*_max_ shifts to longer wavelengths. This is consistent with the emission being dominated by TADF at high temperatures, that is, emission from the singlet excited state, and by phosphorescence at low temperatures, that is, emission from the low energy triplet state. Remarkably, this behavior is not observed for the films of **1** and **2** in l‐PMMA host. Here, the emission comes mainly from the triplet state and the contribution of TADF is practically non‐existent. In this host the vibrational quenching dominates. The emission intensity, therefore, increases with decreasing temperature in the entire temperature range, and more markedly from 320 to 170 K, where the effect of suppressing vibrations is more pronounced. This is presented in Figures 1e and 1i.

In H‐PMMA (Figures 1g and 1h), as in l‐PMMA, the intermolecular interactions between **1** (or **2**) molecules are practically non‐existent. This, therefore, indicates that the H‐PMMA host is more able to suppress vibrations, in the same way as the intermolecular interactions between **1** (or **2**) molecules do in the pristine film. This enables the reverse intersystem crossing to become operative and to activate the TADF emission. However, in l‐PMMA this is not the case, and the internal conversion dominates in the entire temperature range.

Below 170 K the temperature dependence of the photoluminescence is similar in all samples (Figures 1i and 1j). This shows that once *k*_RISC_ is suppressed, and as the effect of vibrations decreases due to lowering the temperature, the differences in the host are not so important to the outcome of the emission. These experiments highlight the fact that the nonradiative pathways play a crucial role in the triplet‐harvesting mechanism in complexes **1** and **2**. Moreover, in the case of **1**, and to lesser extent in **2**, the intermolecular interactions present in the pristine films, due to the molecular packing, are able to effectively suppress vibrations that otherwise will quench TADF.

Time resolved spectroscopy was used to follow the excited state dynamics in **1** and **2**. The luminescence decays of **1** and **2** at 300 K in pristine films and dispersed in l‐PMMA are shown in Figures S7a and S7b. Both luminescence decays of **1** at 300 K are characterized by a fast component around 5 and 7.9 ns in pristine film and l‐PMMA, respectively. These fast components are followed by a long bi‐exponential decay, with time constants of 0.9 and 5 μs in the pristine film, and of 9 and 30 μs in l‐PMMA. The lifetime in l‐PMMA is thus longer than in the pristine film. This reflects the effect of TADF in the pristine film, which at 300 K more rapidly quenches the triplet state, whereas in l‐PMMA the emission occurs mainly directly from the triplet state, for example, phosphorescence. These observations are consistent with the more intense emission in the pristine film below 1 μs, where TADF dominates. Complex **2** shows similar behavior (Figure S7b). Importantly, this reveals also how the presence of TADF accelerates the excited state decay in Cu^I^ complexes and minimizes the roll‐off effect in devices.

The decays obtained as a function of temperature for the pristine films, l‐PMMA and H‐PMMA films, and crystalline samples are entirely consistent with the relative importance of TADF. In pristine films, H‐PMMA and crystalline samples both complexes **1** and **2** exhibit clear temperature dependence for the delayed fluorescence which is characteristic of the TADF mechanism (Figures 2c, 2d and Supporting Information Figure S8). Here with decreasing temperature the emission intensity decreases and the lifetime increases, consistent with a slower depopulation of the triplet state. In contrast, in the l‐PMMA host (Figures 2 a and 2b) the photoluminescence decay is dominated by the nonradiative constants. This is observed in the temperature dependence of the prompt fluorescence component which increases in intensity with decreasing the temperature, and the increase of the phosphorescence lifetime in both compounds, as a direct response of suppressing the nonradiative decay pathways. Note that no TADF component is observed on these decays. In Figures 2e and 2f the power dependence of the delayed fluorescence for both compounds confirms the origin of the mechanism responsible for the long‐lived fluorescence as TADF (slope 1) and not triplet–triplet annihilation (TTA) (slope 2).^10^ These experiments were performed on pristine films, where the TADF component is stronger.

**Figure 2:**
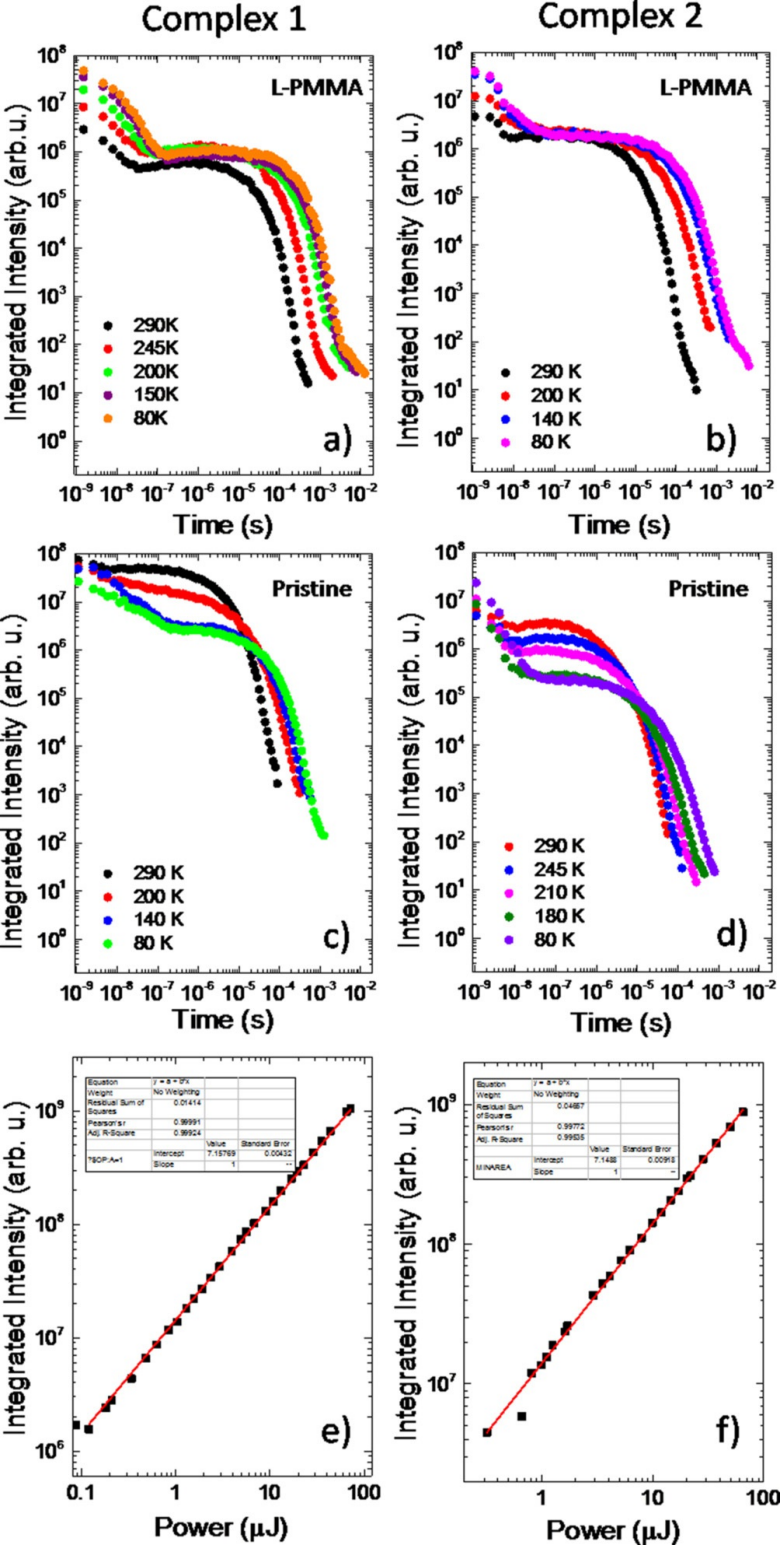
Temperature‐dependence of emission decays in l‐PMMA for a) **1** and b) **2**, respectively. Temperature dependence of emission decays in pristine film for c) **1** and d) **2**, respectively. Power dependence of DF emission in pristine film for e) **1** and f) **2**, respectively.

The two isomeric complexes **1** and **2** differ only in the substitution pattern on the tetrazolate ring, and this is shown to have a profound effect on the supramolecular structure and photophysical properties. It is instructive to compare the single‐crystal X‐ray structures of the two complexes. They both exhibit highly distorted tetragonal coordination, typical of Cu(N N)(POP)^+^ complexes (Figures 3, S10 and S11).6c Crystals of **1** revealed multiple modes of intra‐ and intermolecular interactions leading to a fascinating 3D supramolecular structure (Figure 3). Important interactions are summarized in Table S5. Generally, the presence of strong intra‐ and intermolecular interactions would supply a more rigid environment and thereby effectively limit molecular distortions in the excited state, leading to bright emission through restricting the non‐radiative processes.6d, 11 Furthermore, due to the small Δ*E*(S_1_−T_1_) value obtained from photophysical experiments, and from the DFT calculations (see below), TADF is triggered in the pristine aggregation state of complex **1**, which is similar to the situation in H‐PMMA. It is worth noting that intermolecular aromatic stacking has been shown to offer a charge‐transfer pathway and to enhance the carrier‐transport ability, which is essential for excellent electroluminescence materials.^12^

**Figure 3:**
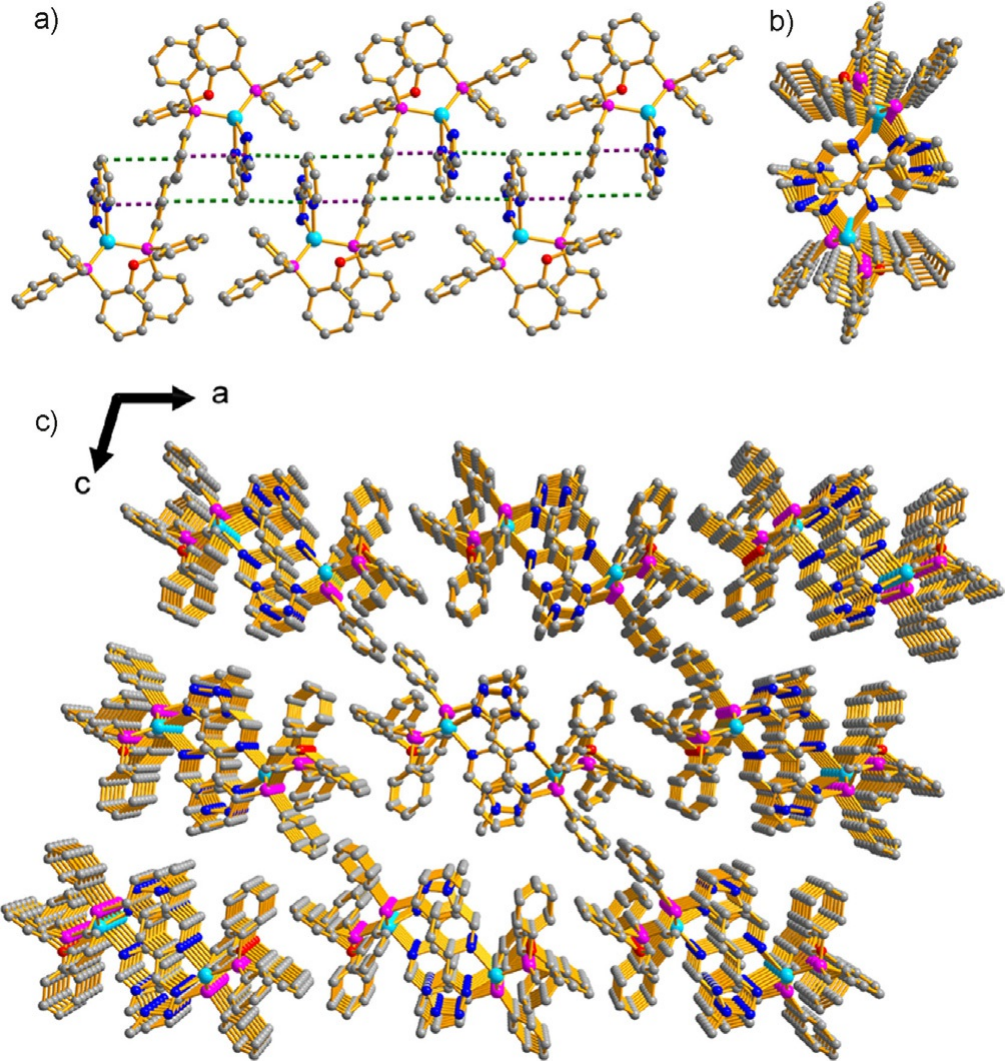
a) The one‐dimensional supramolecular chain structure in **1**. (Purple dashed lines represent intramolecular π⋅⋅⋅π interactions and green dashed lines represent two kinds of intermolecular π⋅⋅⋅π interactions). b) The cross‐section view of the one‐dimensional supramolecular chain structure unit in **1**. c) The three‐dimensional supramolecular structure of **1**. Hydrogen atoms are omitted for clarity.

In the crystal structure of **2**, multiple modes of intra‐ and intermolecular interactions lead to a one‐dimensional supramolecular chain structure (Figure S11).^13^ Due to the relatively weak intermolecular interactions in **2**, compared with **1** (Figure 3) complex **2** showed less distortion of the tetragonal coordination in the ground state. For the excited state, Riesgo et al. stated that the distortion of Cu complexes is directly related to their “Stokes‐like′′ shift, that is, the difference in wavelength between absorption and emission spectra.^14^ Indeed, the smaller Stokes‐like shift for **1** compared with **2** (Figure S6 and Table S1) indicates that excited‐state distortion is suppressed more effectively in **1** which, in turn, means that for **1** the non‐radiative process is suppressed. It could, therefore, be expected that the photoluminescence quantum yield (PLQY) for **1** is substantially higher than **2**. Indeed PLQYs of 47.1 and 9.4 % were measured in the crystalline state for **1** and **2**, respectively (Table S1).

Emission spectra of complexes **1** and **2** in amorphous pristine films and crystalline forms were collected in vacuum and in the presence of oxygen, showing only small variations of the luminescence intensity once oxygen has been removed (Figure S9). This indicates that oxygen quenching is not the cause for the lower PLQY of both complexes in their amorphous films, when compared with their crystalline forms, and in particular for the lower PLQY of complex **2** when compared with complex **1**. Instead, the origin of the lower PLQY in **2** is due to the weaker intermolecular interactions that are prevalent in complex **2** compared with the highly ordered three‐dimensional supramolecular chain structure observed in **1**, but not in **2**.

To further probe the interesting optoelectronic phenomena discussed above, we performed time‐dependent density functional theory (TD‐DFT) calculations on complexes **1** and **2**. The electronic structures based on the optimized geometries of **1** and **2** plotted in Figure S12 show the clearly separate electronic occupations in the HOMO and the LUMO for each complex, facilitating a small Δ*E*(S_1_−T_1_) which is a characteristic of TADF materials.^15^

Since the value of Δ*E*(S_1_−T_1_) is usually used to assess the ability of reverse intersystem crossing from T_1_ to S_1_,^16^ it was evaluated here for every structure (**IM**, **LPM** and **CM**) presented in Figure 4 to explain the change of luminescence properties in the different situations mentioned above. The nomenclature **IM**, **LPM** and **CM** defines the optimized isolated molecule, the simulated molecule in low PMMA (l‐PMMA) host, and the molecule in the crystal structure, respectively. As mentioned above, the complex in l‐PMMA host has more freedom to rotate the phenyl group than in the crystal structure. Therefore, an important dihedral angle θ, which corresponds to the nearest phenyl group to the tetrazole ring, as a representative angle, was changed and scanned for **1** and **2**. The results collected in Table S6 show: (i) both the **IM** and **CM** structures have a smaller Δ*E*(S_1_−T_1_) value, further verifying that TADF may occur in isolated molecules (solution state) and in the aggregation state (crystal structure). However, the huge difference in optimized geometries between ground state and S_1_ along with T_1_ collected in Table S7 also illustrate the large nonradiative decay of single molecules of both complexes in solution. This is crucial for the quenching of TADF in the solution state. (ii) With the increase of θ, Δ*E*(S_1_−T_1_) becomes larger for both **1** and **2**, suggesting the rotation of the nearest phenyl group to the tetrazole ring raises Δ*E*(S_1_−T_1_) relative to the crystal structure, which might greatly reduce the ability for transfer from the T_1_ to the S_1_ state. Thus for **1** and **2** phosphorescent emission, rather than TADF, appears in the l‐PMMA environment where the molecules are free to rotate their phenyl groups. Hence, the relationship between restriction of molecular vibrations and the luminescent properties of the two Cu^I^ complexes are summarized in Figure 4 b.

**Figure 4:**
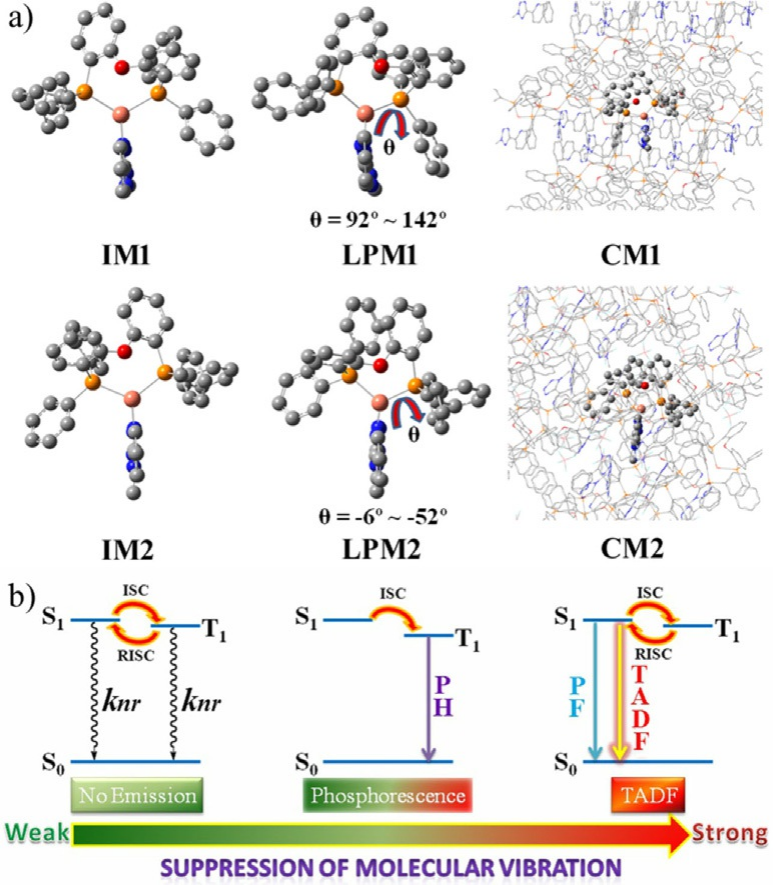
a) Diagram of three models (**IM**, **LPM** and **CM**) for **1** and **2**. The **IM**, **LPM** and **CM** represent the optimized isolated molecule, simulated molecule in low PMMA host material, and the molecule in the crystal structure, respectively. Hydrogen atoms are omitted for clarity. b) The relationship between restriction of molecular vibrations and luminescent properties of the two Cu^I^ complexes.

Moreover, a singlet–triplet gap around 0.2 eV, as predicted in the calculations, is in excellent agreement with the energy difference determined from the onset of the emission spectra in pristine films (see Figure 1 c and 1d).

In summary, we have studied two new AIE–TADF active isomeric Cu^I^ complexes. Solid‐state “dilution” experiments investigated their photophysical properties at different degrees of suppression of molecular vibrations, revealing phosphorescence in low MWt PMMA (l‐PMMA), but efficient TADF in high MWt PMMA (H‐PMMA). The similar TADF behavior in H‐PMMA and in the pristine aggregated state (neat thin films) suggests that TADF behavior is strongly influenced by effectively restricting the vibration of individual molecules through intermolecular interactions. TD‐DFT calculations are in excellent agreement with the observed photophysical properties of the complexes. Our findings may stimulate new molecular engineering endeavours in the design of AIE–TADF active materials with highly emissive aggregated states. Moreover, the extremely short delayed lifetime in pristine film state suggests that both complexes **1** and **2** are candidates for non‐doped OLEDs.

## Conflict of interest

The authors declare no conflict of interest.

## Supporting information

As a service to our authors and readers, this journal provides supporting information supplied by the authors. Such materials are peer reviewed and may be re‐organized for online delivery, but are not copy‐edited or typeset. Technical support issues arising from supporting information (other than missing files) should be addressed to the authors.

Supplementary
