# Supplementary material for: Thermally Activated Delayed Fluorescence in CuI Complexes Originating from Restricted Molecular Vibrations
Source: Chemistry. 2017 Aug 10;23(49):11761–6. doi: 10.1002/chem.201701862 (PMC5724495; doi:10.1002/chem.201701862)
Supplement: Supplementary file 1 — Supplementary [file CHEM-23-11761-s001.pdf]

CHEMISTRY
A European Journal

Supporting Information

Thermally Activated Delayed Fluorescence in Cu^I Complexes Originating from Restricted Molecular Vibrations

Guangfu Li+,[a] Roberto S. Nobuyasu+,[b] Baohua Zhang+,[c] Yun Geng+,[a] Bing Yao,[c] Zhiyuan Xie,[c] Dongxia Zhu,*[a] Guogang Shan,[a] Weilong Che,[a] Likai Yan,[a] Zhongmin Su,[a] Fernando B. Dias,*[b] and Martin R. Bryce*[d]

chem_201701862_sm_miscellaneous_information.pdf

Table of Contents

		Page
I.	Experimental - general information; synthetic details	S3
II.	Photophysical properties	S7
III.	X-ray crystallographic data	S10
IV.	Quantum calculations	S13
V.	Electrochemical data	S15
VI.	References for SI	S16

I. Experimental - general information

Materials obtained from commercial suppliers were used without further purification unless otherwise stated. All glassware, syringes, magnetic stirring bars, and needles were thoroughly dried in a convection oven. Reactions were monitored using thin layer chromatography (TLC). Commercial TLC plates were used and the spots were visualized under UV light at 254 and 365 nm. ^{1}H NMR spectra were recorded at 25 ºC on a Varian 500 MHz spectrometer. The chemical shifts (δ) are given in parts per million relative to internal standard TMS (0 ppm for ^{1}H). ^{31}P NMR spectra were recorded at 25 ºC on a 600 MHz spectrometer. The molecular weights of the complexes were obtained by using matrix-assisted laser desorption-ionization time-of-flight (MALDI-TOF) mass spectrometry. Elemental analysis was obtained using a Flash EA1112 analyzer. UV-vis absorption spectra were recorded on a Shimadzu UV-3100 spectrophotometer. PL efficiencies were measured with an integrating sphere (C-701, Labsphere Inc.), with a 365 nm Ocean Optics LLS-LED as the excitation source, and the laser was introduced into the sphere through the optical fiber. Crystal structure data for complexes **1** and **2** were collected on a Bruker Smart Apex II CCD diffractometer with graphite-monochromated Mo Kα radiation (λ = 0.71073 Å) at room temperature. CCDC numbers 1524132 for **1** and 1524133 for **2**.

Synthesis of (2-pyridyl)tetrazole (L1)

L1 was synthesized according to the literature method.[1] To a 250 mL round-bottomed flask was added the 2-cyanopyridine (20 mmol), sodium azide (1.43 g, 22 mmol), zinc bromide (4.50 g, 20 mmol), and water (40 mL). The reaction mixture was refluxed for 24 h, cooled to room temperature and basified by addition of 2.5 equiv. of NaOH, filtered, acidified by diluted hydrochloric acid to pH = 6.5, and filtered, and the resulting solid was washed with water to give **L1** (2.1 g; 77% yield). ^{1}H NMR (500 MHz, DMSO-d_6, δ [ppm]): 8.79 (m, 1H), 8.22 (d, 1H, J = 9.5 Hz), 8.08 (m, 1H), 7.62 (m, 1H); MS: (MALDI-TOF) [m/z]: 149.06 (M^+) (calcd: for 148.06).

Synthesis of 2-(2-methyl-2*H*-tetrazol-5-yl)pyridine (L2) and 2-(1-methyl-1*H*-tetrazol-5-yl)pyridine (L3)

L2 and **L3** were synthesized according to the literature method.[2] **L1** (1.0 g, 6.8 mmol), iodomethane (3.85 g, 27.1 mmol) and sodium hydroxide (0.68 g, 16.96 mmol) were dissolved in dimethylformamide (15 mL). The mixture was stirred for 24 h at room temperature. Water (100 mL) was added to the solution then extracted with dichloromethane (400 mL). The organic layer was

separated and the mixture was then purified by column chromatography on silica gel (200–300 mesh) with petroleum ether/ethyl acetate (10:3 v/v) to give **L3** (0.52 g, 48% yield) and then using the same solvent system (5:8 v/v) as the eluent to give **L2** (0.316 g, 29% yield). ^{1}H NMR (500 MHz, $CDCl_3$-d_6, δ [ppm]): 8.65 (s, 1H), 8.10 (d, *J* = 7.5 Hz, 1H), 7.74 (t, *J* = 7.5 Hz, 1H), 7.28 (d, *J* = 5.5 Hz, 1H), 4.34 (s, 3H) (**L2**); 8.75 (d, *J* = 4 Hz, 1H), 8.33 (d, *J* = 8.5 Hz, 1H), 7.93 (t, *J* = 7.5 Hz, 1H), 7.48 (m, 1H), 4.52 (s, 3H) (**L3**).

Synthesis of complex 1

Complex **1** was prepared according to the following general procedure: A mixture of $[Cu(CH_3CN)_4]BF_4$ (0.32 g, 1.0 mmol) and POP (0.54 g, 1.0 mmol) in CH_2Cl_2 (10 mL) was stirred at room temperature for 1 h and then the diimine ligand L**2** (0.16 g, 1.0 mmol) was added. The reaction mixture was stirred for another 2 h, and the solvent was then evaporated; the crude product was dissolved in a small amount of dichloromethane and passed through a short column of neutral aluminum oxide. The solvent was removed to obtain complex **1** (0.70 g, 80% yield). ^{1}H NMR (500 MHz, DMSO-d_6, δ [ppm]): 8.48 (d, *J* = 5.0 Hz, 1H), 8.24 (d, *J* = 5.5 Hz, 1H), 8.16 (t, *J* = 9.0 Hz, 1H), 7.62 (t, *J* = 5.5 Hz, 1H), 7.43–7.32 (m, 14H), 7.13–7.08 (m, 12H), 6.74–6.70 (m, 2H), 4.44 (s, 3H). ^{31}P NMR (600 MHz, $CDCl_3$, δ [ppm]): -12.4 (s). MS (MALDI-TOF) [m/z] : 601.1 (1-BF_4–-L**2**). Anal. Calcd for $C_{43}H_{35}BCuF_4N_5OP_2$: C, 60.76; H, 4.15; N, 8.24. Found: C, 60.51; H, 4.23; N, 8.33. Crystals for X-ray analysis were grown by slow evaporation of a solution of complex **1** in CH_2Cl_2 and MeOH mixture.

Synthesis of complex 2

The synthetic procedure of complex **2** was the same as complex **1**, except ligand L**3** (0.16 g, 1.0 mmol) was used instead of L**2**. Yield: 0.72 g, 82%. ^{1}H NMR (500 MHz, DMSO-d_6, δ [ppm]): 8.48 (d, *J* = 5.0 Hz, 1H), 8.24 (d, *J* = 5.5 Hz, 1H), 8.16 (t, *J* = 9.0 Hz, 1H), 7.62 (t, *J* = 5.5 Hz, 1H), 7.43–7.32 (m, 14H), 7.13–7.08 (m, 12H), 6.74–6.70 (m, 2H), 4.44 (s, 3H). ^{31}P NMR (600 MHz, $CDCl_3$, δ [ppm]): -12.0 (s). MS (MALDI-TOF) [m/z] : 601.1 (**2**-BF_4^--L**3**). Anal. Calcd for $C_{43}H_{35}BCuF_4N_5OP_2$: C, 60.76; H, 4.15; N, 8.24. Found: C, 60.45; H, 4.18; N, 8.17. Crystals for X-ray analysis were grown by slow evaporation of a solution of complex **2** in CH_2Cl_2 and MeOH mixture.

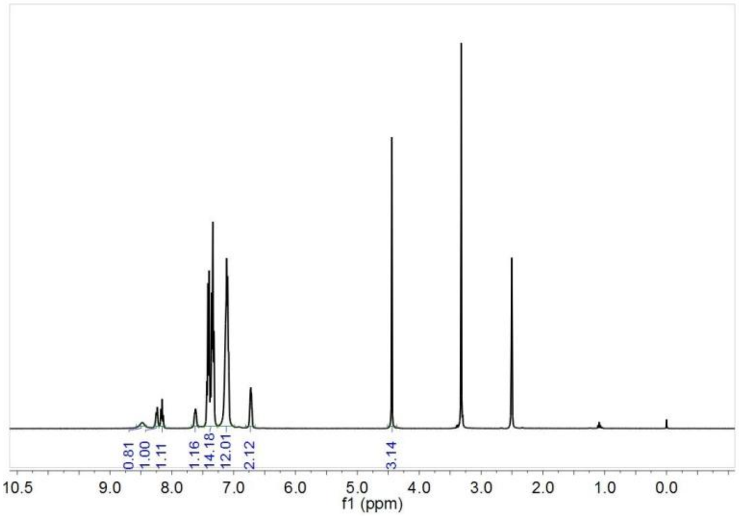

Figure S1. ^{1}H NMR spectrum of complex **1**.

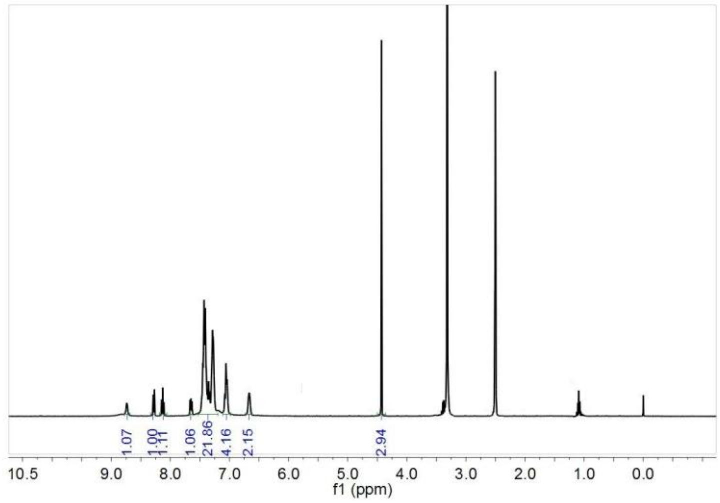

Figure S2. ^{1}H NMR spectrum of complex **2**.

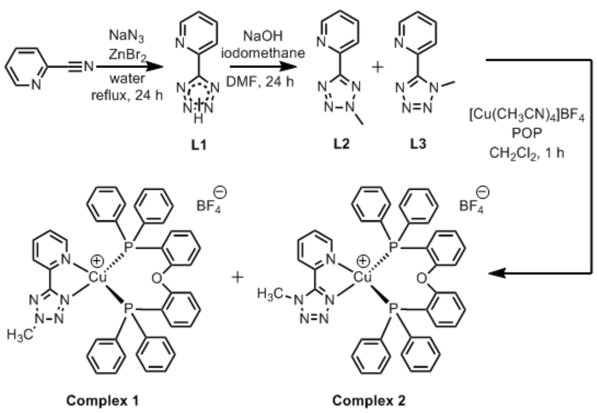

Scheme S1. Synthesis of L**1**, L**2**, L**3**, complex **1** and complex **2**.

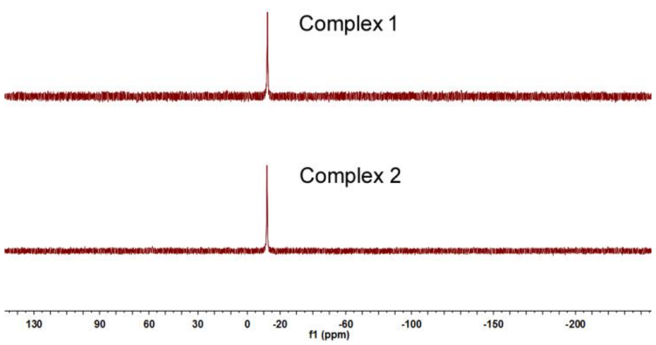

Figure S3. ^{31}P NMR spectra of complex **1** and complex **2**.

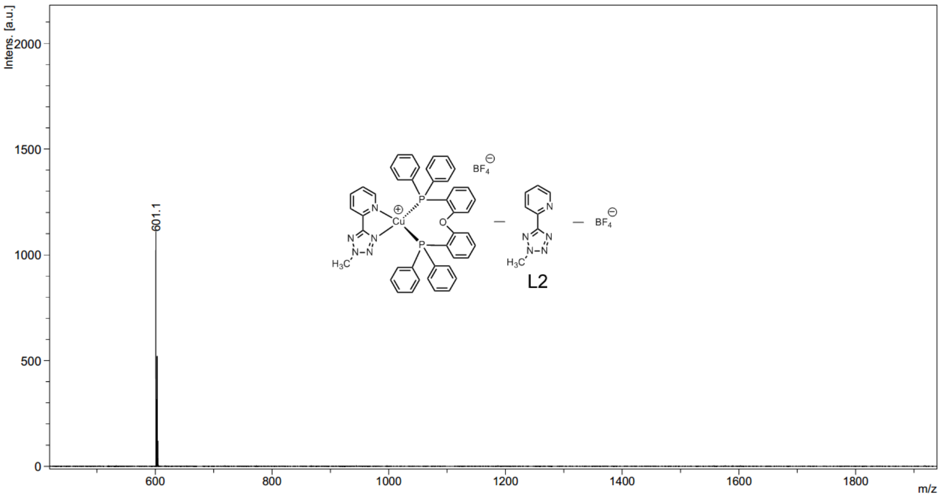

Figure S4. MALDI-TOF spectrum of complex **1**.

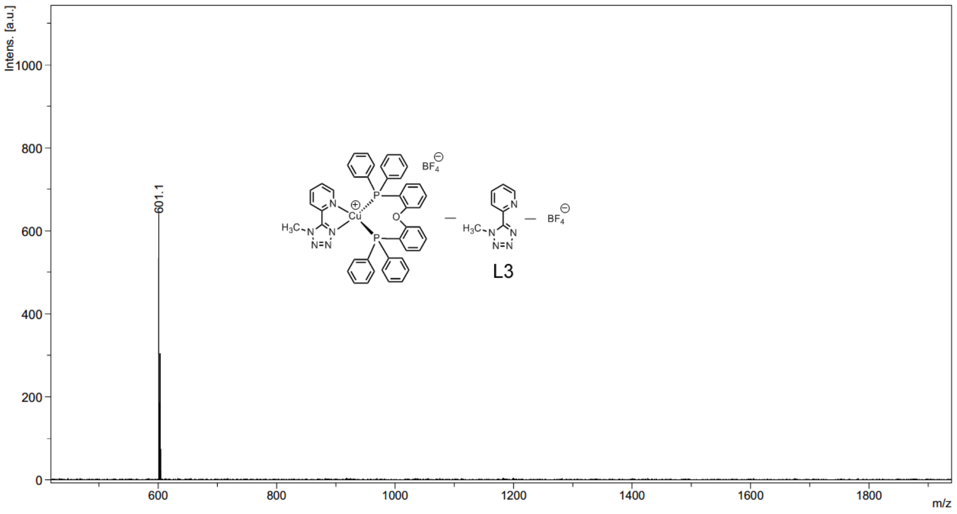

Figure S5. MALDI-TOF spectrum of complex **2**.

II. Photophysical properties

Photophysical Characterization. Thin films of Cu(I) complexes **1** and **2** in a matrix were prepared by spin-coating a blend of complex : matrix (1:20 w/w). L-PMMA (average Mw ~15,000) and H-PMMA (average Mw ~960,000) were obtained from Sigma-Aldrich. Thin films of pristine Cu(I) complexes were prepared by spin-coating from DCM solutions (10 mg/mL). Steady-state photoluminescence data were collected using a Fluorolog fluorescence spectrometer (Jobin Yvon). Temperature dependence studies were performed using a cryostat with a liquid nitrogen cooling system. Phosphorescence, prompt fluorescence (PF), delayed fluorescence (DF) and decays were recorded using nanosecond gated luminescence and lifetime measurements (from 1 ns to 1 s) with either a high energy pulsed Nd:YAG laser emitting at 355 nm (EKSPLA) or a N2 laser emitting at 337 nm. Emission was focused into a spectrograph and detected on a sensitive gated iCCD camera (Stanford Computer Optics) having a sub-nanosecond resolution. PF/DF time resolved measurements were performed by increasing exponentially the gate and the delay times.

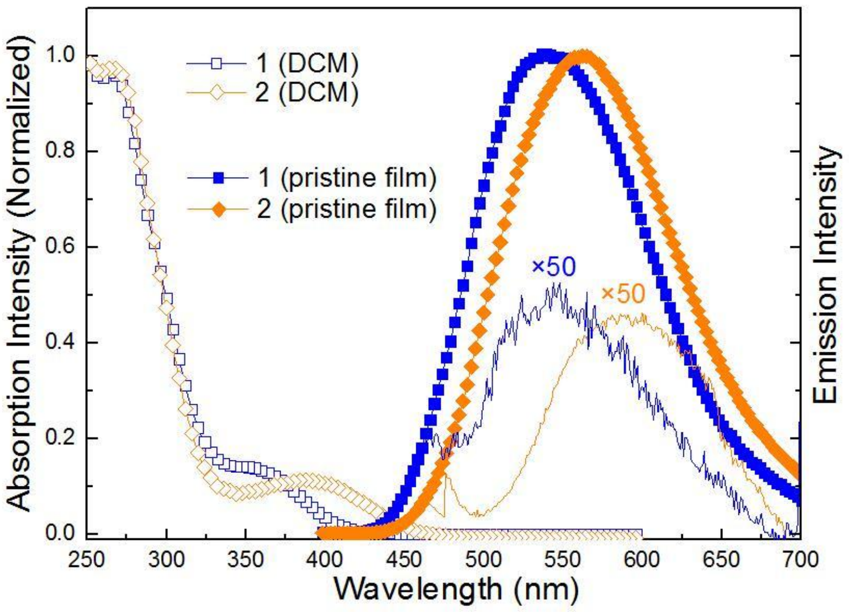

Figure S6. UV/vis absorption and emission spectra of **1** and **2** in degassed solution (CH_2Cl_2) and pristine film at room temperature.

Table S1. Photophysical data for complexes 1 and 2 at room temperature

Complex	$\lambda_{abs}{}^{a}$ (nm)	$\lambda_{em}{}^{b,c,d}$ (nm)	τ^{b} (μs)	$\Phi_{L}{}^{b,e}$ (%)
**1**	264, 358_{sh}	533, 516, 516	0.90, 5.0	15.5, 47.1
**2**	269, 386	572, 556, 556	0.95, 4.2	2.9, 9.4

[a] Measured in CH_2Cl_2 (1.0×10^{-5} M). [b] Measured in the pristine film; error ± 5%. [c] Measured in low molecular weight (L-PMMA) film. [d] Measured in the high molecular weight (H-PMMA) film. [e] Measured in the crystalline state.

Table S2. Energy levels and S_1-T_1 gaps of the complexes 1 and 2

Complex	$\Delta E_g{}^{a}$ (eV)	HOMO[b] (eV)	LUMO[c] (eV)	$S_1{}^{d}$ (eV)	$T_1{}^{e}$ (eV)	ΔE_{ST} (eV)
**1**	3.0	-5.5	-2.5	2.75	2.583	0.167
**2**	2.72	-5.49	-2.77	2.719	2.50	0.219

[a] Estimated from the onset wavelengths of the absorption spectra measured in CH_2Cl_2. [b] Calculated from the oxidation potentials obtained by cyclic voltammetry. [c] Calculated from the HOMO energy levels and E_g. [d] Estimated from the onset wavelengths of the 298 K emission spectra measured as pristine film. [e] Estimated from the onset wavelengths of the 77 K emission spectra measured as pristine film.

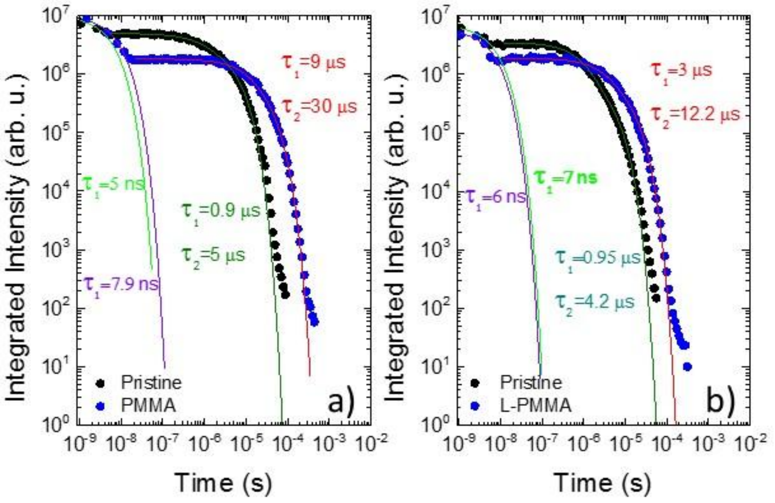

Figure S7. The fluorescence decays collected in pristine and L-PMMA films at room temperature for complexes **1** a) and **2** b), respectively.

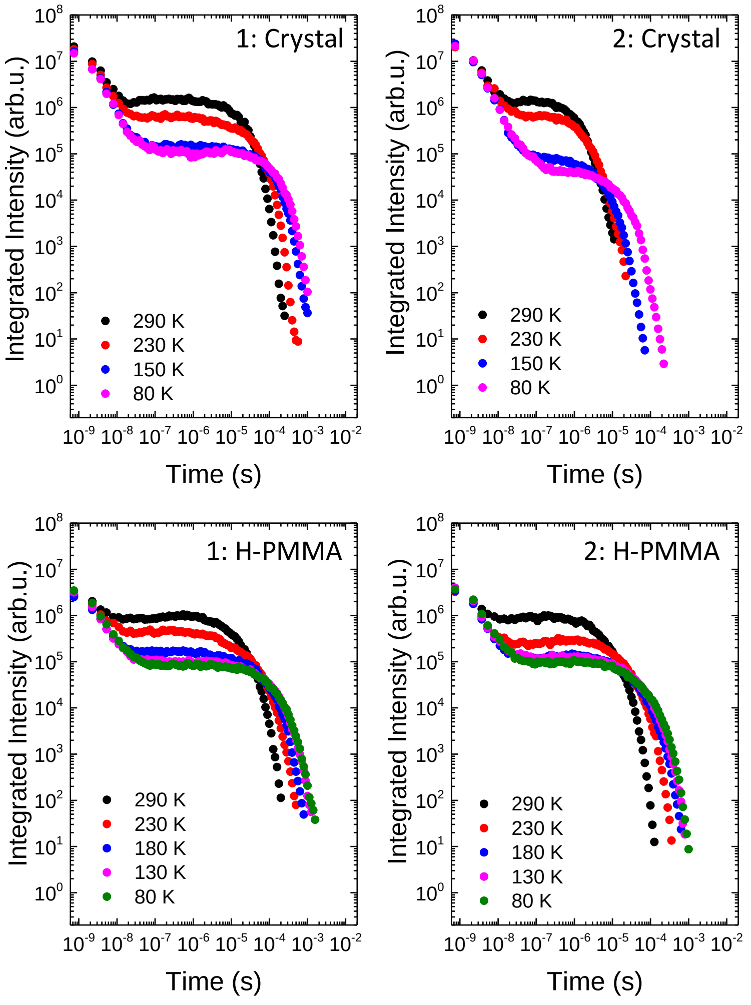

Figure S8. Temperature dependence of emission decays of crystals and H-PMMA films of **1** and **2**.

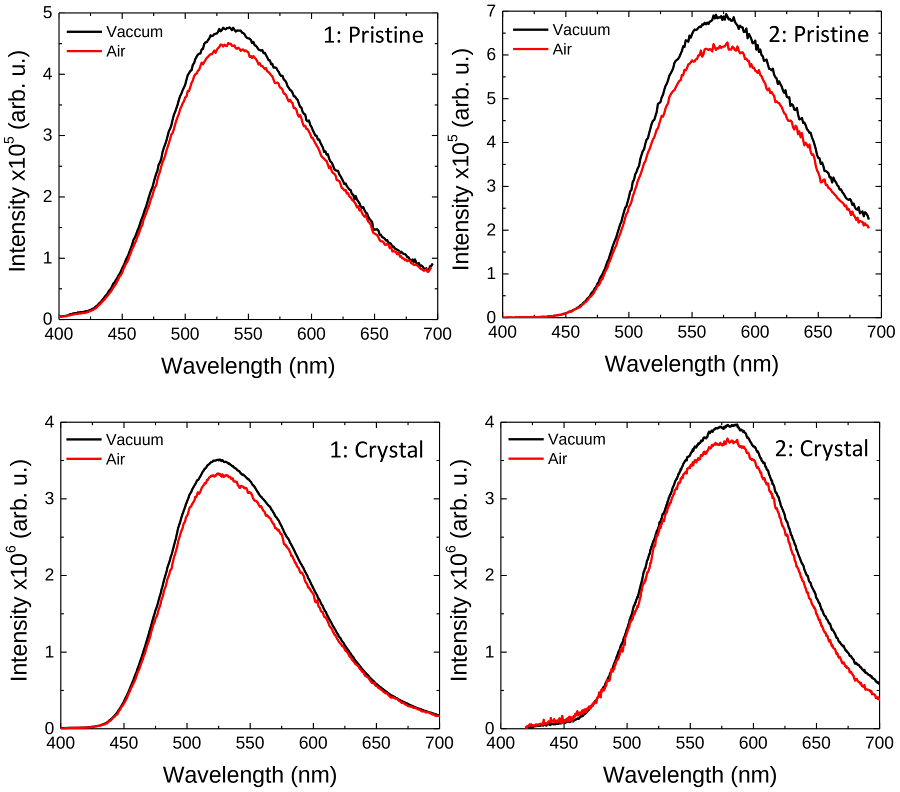

Figure S9. Steady state photoluminescence spectra under vacuum and in air, for complexes **1** and **2** in pristine film and in crystal.

III. X-ray crystallographic data

The molecular structures of complexes **1** and **2** were confirmed by X-ray crystallographic analysis of single crystals. Diffraction data were collected on a Bruker SMART Apex CCD diffractometer using k(Mo-Kα) radiation (k = 0.71073 Å). Cell refinement and data reduction were made by the SAINT program. The structure was determined using the SHELXTL/PC program. Figure S10 shows Oak Ridge thermal ellipsoid plot (ORTEP) drawings of complexes **1** and **2**. The crystallographic data have been deposited with the Cambridge Crystallographic Data Centre with CCDC deposition numbers 1524132 and 1524133. These data can be obtained free of charge from The Cambridge Crystallographic Data Centre via www.ccdc.cam.ac.uk/data_request/cif.

Table S3. Crystal data and structure refinement for 1

	**1**
Empirical formula	$C_{43}H_{35}BCuF_4N_5OP_2$
Formula weight	850.05
Temperature (K)	293(2)
Crystal system	Monoclinic
space group	P2(1)/c
a /Å	16.358(3)
b /Å	11.479(2)
c /Å	23.685(5)
α /°	90.00
β /°	106.59(3)
γ /°	90
V/Å^3	4262.5(15)
Z	4
ρ_{calc} (g/cm^3)	1.325
μ/mm^{-1}	0.644
R_{int}	0.0623
Goodness-of-fit on F^2	1.007
R_1^a, wR_2^b [I>2σ(I)]	0.0702, 0.1866
R_1, wR_2 (all data)	0.1113, 0.2074

^{a} $R_1 = \Sigma||Fo| - |Fc||/\Sigma|Fo|$. ^{b} $wR_2 = \{ \Sigma[w(Fo^2 - Fc^2)^2]/ \Sigma[w(Fo^2)^2]\}^{1/2}$

Table S4. Crystal data and structure refinement for 2

	**2**
Empirical formula	$C_{43}H_{35}BCuF_4N_5OP_2$
Formula weight	850.05
Temperature (K)	296(2)
Crystal system	Orthorhombic
space group	Fddd
a /Å	25.1061(14)
b /Å	30.1001(14)
c /Å	48.205(3)

α /°	90.00
β /°	90
γ /°	90
V/Å^3	36428(4)
Z	32
ρ_{calc} (g/cm^3)	1.240
μ/mm^{-1}	0.603
R_{int}	0.1324
Goodness-of-fit on F^2	0.977
R_1^{a}, wR_2^{b} [I>2σ(I)]	0.0786, 0.2027
R_1, wR_2 (all data)	0.1661, 0.2584

^{a} $R_1 = \Sigma||Fo| - |Fc||/\Sigma|Fo|$. ^{b} $wR_2 = \{ \Sigma[w(Fo^2 - Fc^2)^2]/ \Sigma[w(Fo^2)^2]\}^{1/2}$

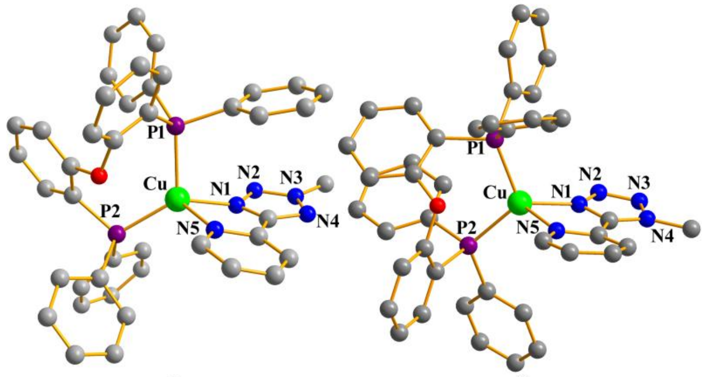

1 **2**

Figure S10. ORTEP drawings of **1** and **2**. The BF_4 counterion, H atoms and solvent molecules have been omitted for clarity. Selected bond lengths (Å) and angles (º): For **1** Cu–N1 2.059(4), Cu–N5 2.120(5), Cu–P1 2.280(14), Cu–P2 2.2136(14); N1–Cu–N5 79.09(18), P1–Cu–P2 119.92(5), N1–Cu–P1 101.63(13), N1–Cu–P2 129.26(13), N5–Cu–P1 105.41(12), N5–Cu–P2 112.82(13). For **2** Cu–N1 2.041(5), Cu–N5 2.098(6), Cu–P1 2.2480(19), Cu–P2 2.2381(18); N1–Cu–N5 79.2(2), P1–Cu–P2 113.63(7), N1–Cu–P1 118.14(17), N1–Cu–P2 118.03(16), N5–Cu–P1 107.78(16), N5–Cu–P2 114.76(16) .

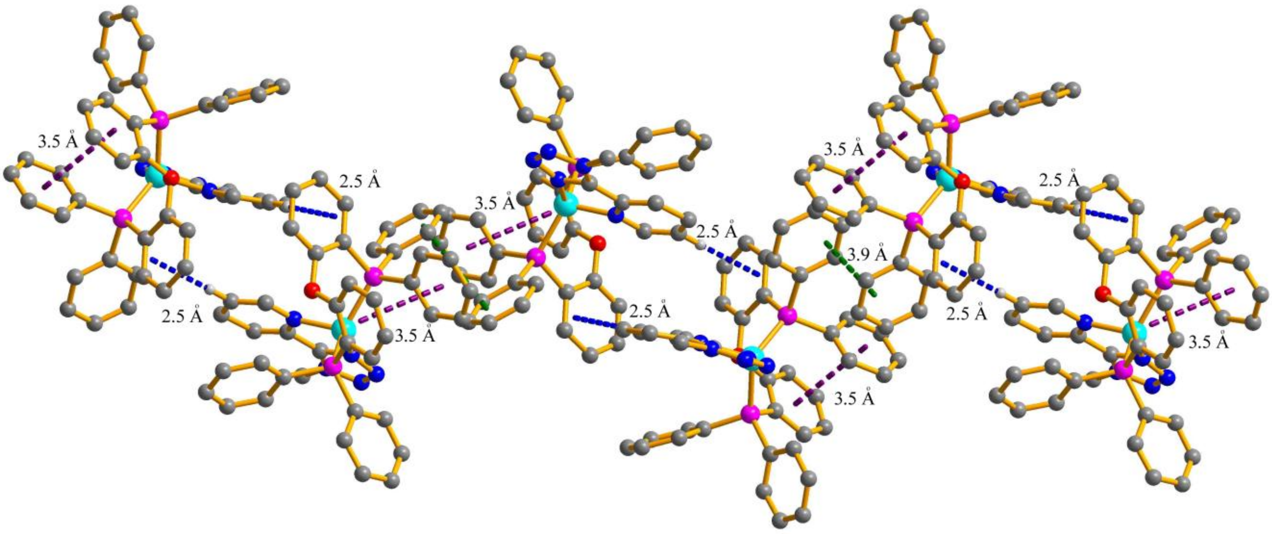

Figure S11. The one-dimensional supramolecular chain structure unit in **2** (Purple dashed lines represent intramolecular π···π interactions, blue dashed lines represent intermolecular C-H···π interactions and green dashed lines represent intermolecular π···π interactions).

Table S5. The distances of intra- or intermolecular interactions for 1 and 2

1		2	
Intramolecular π···π stacking	3.6 Å	Intramolecular π···π stacking	3.5 Å
Intermolecular π···π stacking	3.5 Å	Intermolecular C-H···π interaction	2.5 Å
Intermolecular head-to-tail stacking	3.7 Å	Intermolecular head-to-tail stacking	3.9 Å

IV. Quantum calculations

The single molecule geometries in the ground state and the first triplet excited state (T_1) were optimized by employing the B3LYP exchange-correlation functional. The optimal geometries of the first singlet excited state (S_1) and the energy gap between S_1 and T_1, namely $\Delta E(S_1\text{-}T_1)$, were obtained using TDDFT with the B3LYP functional. For all the calculations, the basis set 6-31G(d,p) was adopted for non-metallic atoms, O, N, C, P, H atoms, and the basis set LAND2DZ was applied on Cu atoms. The Gaussion 09 software was used for all calculations.

LUMO

HOMO

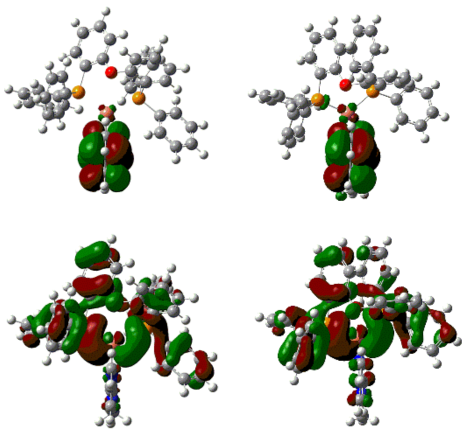

1 2

Figure S12. The frontier molecular orbitals of **1** and **2** based on optimized ground-state geometries calculated at B3LYP/6-31G(d,p) (LAND2DZ for Cu atoms) level.

Table S6. The ΔE(S_1-T_1) (eV) values of three models (IM, LPM and CM) for 1 and 2 obtained by B3LYP/6-31G(d,p) (LAND2DZ for Cu atoms) method. For a definition of θ see Figure 4.

	1			2	
**IM 1**	0.136		**IM 2**	0.177	
	θ = 92	0.185		θ = -6	0.271
	θ = 97	0.202		θ = -11	0.277
	θ = 102	0.224		θ = -16	0.283
	θ = 107	0.248		θ = -21	0.290
	θ = 112	0.271		θ = -26	0.297
**LPM 1**	θ = 117	0.291	**LPM 2**	θ = -31	0.303
	θ = 122	0.307		θ = -36	0.308
	θ = 127	0.319		θ = -41	0.311
	θ = 132	0.326		θ = -46	0.314
	θ = 137	0.325		θ = -51	0.315
	θ = 142	0.316		θ = -56	0.315
**CM 1**	0.185		**CM 2**	0.271	

Table S7. The optimized bond lengths (Å), bond angles (°) and dihedral angles (°) of 1 and 2 at ground (S_0) state and S_1 state along with T_1 state

	**1**				**2**		
	S_0	S_1	T_1		S_0	S_1	T_1
Cu1-P2	2.379	2.408	2.433	Cu1-P2	2.355	2.465	2.447
Cu1-P3	2.363	2.487	2.456	Cu1-P3	2.379	2.456	2.444
Cu1-N5	2.202	2.169	2.074	Cu1-N5	2.158	2.087	2.022
Cu1-N9	2.215	2.059	1.990	Cu1-N9	2.241	2.119	2.028
P2-Cu1-N9	113.5	143.4	141.8	P2-Cu1-N9	114.0	101.8	101.7
P3-Cu2-N5	119.5	123.1	128.1	P3-Cu2-N5	108.0	96.8	97.0
C20-P2-Cu1-N9	166	159.5	164.9	C20-P2-Cu1-N9	-24.3	-29.4	-26.0
C21-C20-P2-Cu1	-34.1	-38.5	-44.8	C21-C20-P2-Cu1	-123.6	-116.3	-114.0

V. Electrochemical data

Cyclic voltammetry was performed in a gas-tight single-compartment three-electrode cell with a BAS Epsilon Electrochemical Analyzer at room temperature. A glassy carbon disk and a platinum wire were the working and auxiliary electrodes, respectively. The reference electrode was Ag/Ag^+ (0.1 M $AgNO_3$ in acetonitrile). The CV measurements were carried out in anhydrous and nitrogen-saturated acetonitrile solutions with 0.1 M *n*-tetrabutylammonium perchlorate (TBAP) and 1.0 mM cuprous complex. The ferrocenium/ferrocene couple was used as an internal standard.

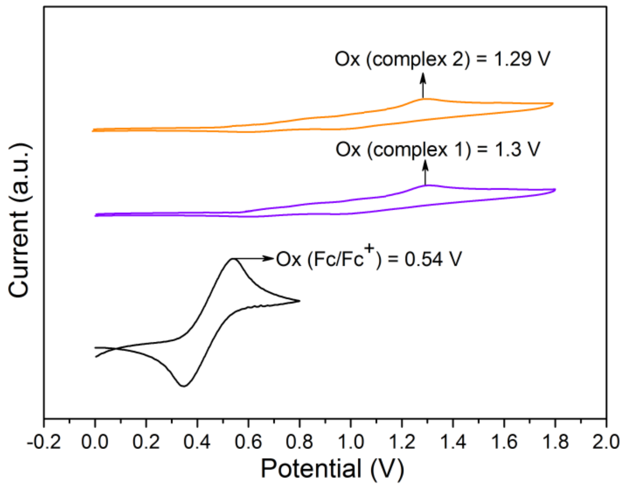

Figure S13. Cyclic voltammograms of ferrocenium/ferrocene couple (as an internal standard) and complexes **1** and **2** measured in acetonitrile in the presence of 0.1 M *n*-tetrabutylammonium perchlorate (TBAP) as supporting electrolyte with 100 mV/s scan speed.

VI. References for SI

(1) Z. P. Demko, K. B. Sharpless, *J. Org. Chem.* **2001**, *66*, 7945-7950.

(2) A. J. Downard, P. J. Steel, J. Steenwijk, *Aust. J. Chem.* **1995**, *48*, 1625-1642.
